# The quest for load-independent left ventricular chamber properties: Exploring the normalized pressure phase plane

**DOI:** 10.1002/phy2.43

**Published:** 2013-08-22

**Authors:** Erina Ghosh, Sándor J Kovács

**Affiliations:** 1 Cardiovascular Biophysics Laboratory, Cardiovascular Division, Department of Internal Medicine, Washington University School of MedicineSt. Louis, Missouri; 2 Department of Biomedical Engineering, School of Engineering and Applied Science, Washington University in St. LouisSt. Louis, Missouri

**Keywords:** Catheterization, diastolic function, hemodynamics, phase plane analysis

## Abstract

The pressure phase plane (*PPP*), defined by dP(t)/dt versus P(t) coordinates has revealed novel physiologic relationships not readily obtainable from conventional, time domain analysis of left ventricular pressure (LVP). We extend the methodology by introducing the normalized pressure phase plane (*nPPP*), defined by 0 ≤ *P* ≤ 1 and −1 ≤ *dP/dt* ≤ +1. Normalization eliminates load-dependent effects facilitating comparison of conserved features of *nPPP* loops. Hence, insight into load-invariant systolic and diastolic chamber properties and their coupling to load can be obtained. To demonstrate utility, high-fidelity P(t) data from 14 subjects (4234 beats) was analyzed. P_NR_, the *nPPP* (dimensionless) pressure, where –dP/dt_peak_ occurs, was 0.61 and had limited variance (7%). The relative load independence of P_NR_ was corroborated by comparison of *PPP* and *nPPP* features of normal sinus rhythm (NSR) and (ejecting and nonejecting) premature ventricular contraction (PVC) beats. PVCs had lower P(t)_max_ and lower peak negative and positive dP(t)/dt values versus NSR beats. In the *nPPP*, +dP/dt_peak_ occurred at higher (dimensionless) P in PVC beats than in regular beats (0.44 in NSR vs. 0.48 in PVC). However, P_NR_ for PVC versus NSR remained unaltered (P_NR_ = 0.64; *P* > 0.05). Possible mechanistic explanation includes a (near) load-independent (constant) ratio of maximum cross-bridge uncoupling rate to instantaneous wall stress. Hence, *nPPP* analysis reveals LV properties obscured by load and by conventional temporal P(t) and dP(t)/dt analysis. *nPPP* identifies chamber properties deserving molecular and cellular physiologic explanation.

## Introduction

The gold standard for characterization of chamber properties utilizes high-fidelity, micromanometric left ventricular (LV) pressures (P) as a function of time. The usual parameters include: maximum and minimum LV pressures (P_max_ and P_min_), peak positive and peak negative rate of change of pressure (+dP/dt_peak_ and –dP/dt_peak_), diastatic pressure, and end-diastolic pressure (EDP). For isovolumic relaxation (IVR) characterization, P from just after –dP/dt_peak_ to just before mitral valve opening is fit using a 2 or 3 parameter assumed exponential relationship (Weiss et al. 1976) which includes the time constant of isovolumic relaxation τ (Matsubara et al. 1995). LVP during the remaining >95% of the cardiac cycle is usually not analyzed.

Eucker et al. (2001) adopted the phase plane analysis method familiar in nonlinear dynamics (Strogatz 2008) to analyze LVP in the pressure phase plane (*PPP)* (Eucker et al. 2002). The oscillatory nature of P during the cardiac cycle generates closed *PPP* loops (analogs of limit cycles) allowing visualization of dP/dt versus P relation especially during the isovolumic phases when dP/dt reaches its respective systolic and diastolic maxima. *PPP* analysis has been used to characterize LV relaxation using various mathematical assumptions (Leite-Moreira et al. 1999; Chung and Kovács 2007, 2008; Senzaki and Kass 2010). Senzaki and Kass (2010) fit the IVR segment in the *PPP* using a logistic model (parameter τ_L_) and showed that it provides a better fit to curved segments than the linear fit (τ) provided by the exponential model. *PPP* analysis of IVR has also led to a predictive, causal kinematic model, where P(t) is the solution to the equation of motion of a damped oscillator (three parameters) allowing for fit of the model predicted solution from before –dP/dt_peak_ to MVO (Chung and Kovács 2008). *PPP* analysis provides a way to visualize spatiotemporal differences in LV hemodynamics (Ghosh and Kovács 2012) during IVR. It has also led to the development of a load independent index of IVR (Shmuylovich and Kovács 2008). Here, we extend *PPP* analysis and introduce the normalized pressure phase plane (*nPPP*) defined by *−1 ≤ dP/dt ≤ 1, 0 ≤ P ≤ 1*. Thus normalization eliminates load-dependent components of P and dP/dt and retains intrinsic contraction and relaxation features of the loops and helps to elucidate and characterize their differences and similarities.

Specifically, we focus on the values of normalized (dimensionless) pressure during isovolumic contraction (IVC) at +dP/dt_peak_ (P_NC_) and during IVR at −dP/dt_peak_ (P_NR_). Additionally, we analyze loops of normal sinus rhythm (NSR) and premature ventricular contraction (PVC) beats within and among subjects. In our exploration of the normalized pressure phase plane we hypothesize that *nPPP* analysis will elucidate novel chamber properties.

## Method

### Derivation of normalized P and dP/dt contours

**Figure 1:**
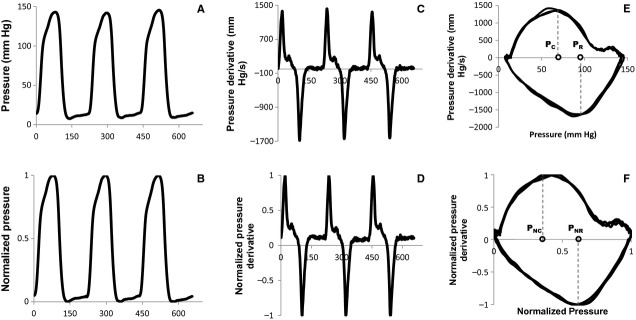
Method for converting P and dP/dt into normalized contours and creating *PPP* and n*PPP* shown in two beats. P_C_ and P_R_ are marked in E and P_NC_ and P_NR_ are marked in F. See text for details.

For each cardiac cycle, LVP was normalized according to:


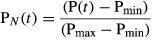
(1)

which assures that P_min_ = 0 and P_max_ = 1. Figure 1A and B illustrate three beats before and after normalization.

The LV dP/dt was normalized according


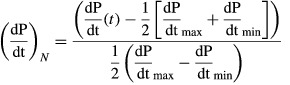
(2)

yielding –dP/dt_peak_ = −1 and +dP/dt_peak_ = +1 for each beat. Results are illustrated in Figure 1C and D with normalized loops in Figure 1E and F.

### Inclusion criteria and data acquisition

We analyzed 17 datasets from our Cardiovascular Biophysics Laboratory database of simultaneous echocardiographic and high-fidelity hemodynamic recordings. Group clinical characteristics are listed in Table 1 (14 subjects) and Table 2 (three subjects). Prior to data acquisition, each subject provided signed, informed consent for participation in accordance with the Institutional Review Board (Human Research Protection Office) of Washington University School of Medicine. The criteria for data selection included: normal LV ejection fraction, normal sinus rhythm, absence of valvular abnormalities and the absence of wall-motion abnormalities or bundle branch block on the ECG. None of the subjects (in Table 1) had a history of coronary artery disease or myocardial infarction. Subjects in the PVC analysis part of the study were selected from the database using the criterion that they had a significant number of PVC beats to enable statistical analysis. One subject in the PVC analysis group (Table 2) had a history of coronary artery disease/myocardial infarction and low ejection fraction. All patients underwent elective cardiac catheterization at the request of a referring cardiologist to establish the presence or absence of suspected coronary artery disease.

**Table 1:** Subject demographics (n = 14)

Parameter	Mean ± SD
Age (years)	62 ± 9
Gender	7M/7F
Height (cm)	167 ± 9
Weight (lb)	182 ± 43
BMI (kg/m^2^)	29.7 ± 7.8
EDP (mm Hg)	18 ± 3
ESP (mm Hg)	105 ± 7
Ejection fraction (%)	72 ± 8
No. of beats	302 ± 43
Hypertension	7 (50%)

Values are mean ± standard deviation or number (% of total subjects).

**Table 2:** Subject demographics for intrasubject (n = 3) PVC analysis

	Subject B1	Subject B2	Subject B3
Age (years)	43	63	56
Gender	M	M	M
Ejection fraction (%)	81	24	54
Height (cm)	196	183	170
Weight (lb)	335	206	165
BMI (kg/m^2^)	40	28	26
EDP (mm Hg)	17	15	10
Hypertension	+	+	−
CAD/previous MI	−	+	−
Total NSR beats	150	232	210
Total PVC beats	9 (NE)	78 (E)	17 (E)

+/– denote presence or absence of condition. NE, nonejecting PVC; E, ejecting PVC.

Our method of high-fidelity, multichannel micromanometric LVP and simultaneous echocardiography recording has been previously detailed (Chung and Kovács 2008; Shmuylovich and Kovács 2008; Ghosh and Kovács 2012). Briefly, simultaneous LV pressure and aortic root pressure measurements were obtained using a 6-F triple transducer pigtail-tipped pressure–volume conductance catheter (SSD-1034; Millar Instruments, Houston, TX). The signal was amplified and calibrated via standard transducer control units (TC-510; Millar Instruments). Catheter placement was achieved by using fluoroscopy to cross the aortic valve, noting that both (distal and mid) pressure channels displayed LV pressure waveforms while the proximal (3rd) sensor displayed aortic root pressures. Pressure signals were input to clinical monitoring systems (Quinton Diagnostics, Bothell, WA or GE Healthcare, Milwaukee, WI) and a custom personal computer via a research interface (Sigma-5DF; CD Leycom, Zoetermeer, The Netherlands) at a sampling rate of 250 Hz. Conductance signals were stored but were not used in this study. Ejection fraction was computed from the calibrated ventriculogram (33 mL of contrast at 11 mL/sec, via 6F pigtail catheter (Cordis Corporation, NJ) immediately after hemodynamic recording.

### Hemodynamic data analysis

Pressure was converted for analysis via a custom Matlab script (Matlab 6.0; MathWorks, Natick, MA). Data sets were smoothed digitally by using a five-point average to suppress noise in the derivative (Shmuylovich and Kovács 2008; Ghosh and Kovács 2012), attenuating 50% of signal at 40 Hz and 90% above 60 Hz, followed by calculation of continuous dP/dt versus time t from the smoothed data. For each beat, EDP and –dP/dt_peak_ were extracted from *PPP* or equivalent time domain contours for both pressure signals.

From the *nPPP*, the P_NC_ and P_NR_ were obtained as shown in Figure 1 using a custom MATLAB script. For all subjects, the mean value of P_max_, P_min_ +dP/dt_peak_, and –dP/dt_peak_, pressure at +dP/dt_peak_ (P_C_), pressure at –dP/dt_peak_ (P_R_), P_NC_ and P_NR_ were calculated and saved.

**Figure 2:**
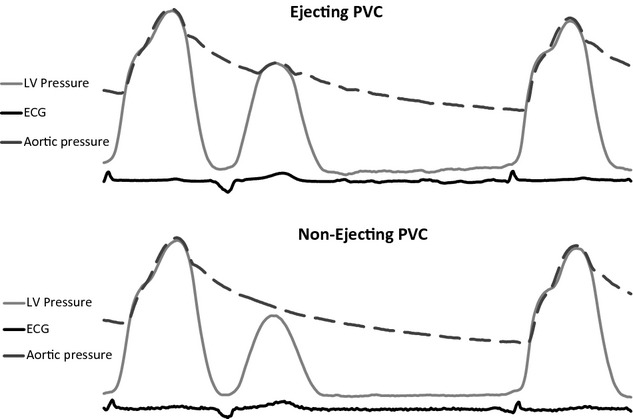
Raw hemodynamic data illustrating criteria by which ejecting and nonejecting PVCs were identified. (Top) Ejecting PVC transiently exceeds aortic root pressure. (Bottom) Nonejecting PVC does not exceed aortic root pressure. See text for details.

We selected three subjects' datasets for PVC analysis. PVCs were first identified from ECG recordings and then classified as ejecting PVCs (E-PVC) or nonejecting PVCs (NE-PVC) by comparing LVP to the simultaneous aortic root pressure. If the LV pressure and the aortic root pressure recordings intersected and the aortic pressure showed a rise and fall concordant with the LV pressure it was classified as E-PVC otherwise it was classified as NE-PVC as shown in Fig 2. For the subjects the mean value of P_max_, P_min_, +dP/dt_peak_, –dP/dt_peak_, P_C_, and P_R_ were calculated for NSR beats as well as E-PVC beats and NE-PVC beats. Mean values for P_NC_ and P_NR_ in NSR, E-PVC and NE-PVC beats were calculated.

### Statistical analysis

The mean, standard deviation (SD), maximum and minimum values were calculated for the points of interest in the regular *PPP* and n*PPP*. In addition, to determine variation we calculated the coefficient of variation, defined as the ratio of standard deviation to the mean value of the parameter, (as shown in Table 3) expressed as a percentage for the 14 subjects included in the intersubject analysis. To compare NSR and PVC features we used the Student's two-tailed t-test to determine statistical significance, with *P* < 0.05 denoting significance.

**Table 3:** Group values (n = 14) of hemodynamic parameters

Parameter	Mean	SD	Min	Max	Variation
P_max_ (mm Hg)	137	17	119	165	12.1%
P_min_ (mm Hg)	9.3	3	3.6	13.4	29.7%
dP/dt_max_ (mm Hg/sec)	1257	136	1062	1531	10.8%
dP/dt_min_ (mm Hg/sec)	−1496	182	−1834	−1266	12.2%
P_C_ (mm Hg)	61	6	49	72	9.6%
P_R_ (mm Hg)	87	10	75	104	11.3%
P_NC_ (dimensionless)	0.41	0.04	0.34	0.48	10.5%
P_NR_ (dimensionless)	0.61	0.04	0.56	0.69	6.6%
EDP (mm Hg)	18	3	13	23	17.9%
ESP (mm Hg)	105	7	93	116	6.9%
nEDP (dimensionless)	0.07	0.02	0.03	0.1	27.1%
nESP (dimensionless)	0.76	0.07	0.64	0.9	8.9%

Coefficient of variation is defined in Methods. SD, Standard deviation.

## Results

**Figure 3:**
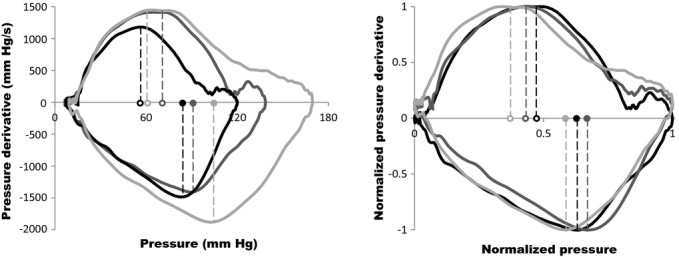
(Left) *PPP* in three subjects. The open circles represent P_C_ and the closed circles represent P_R_. (Right) n*PPP* for the same beats with the open circles representing P_NC_ and closed circles representing P_NR_. See text for details.

Table 1 shows the subject characteristics for the 14 subjects. Table 3 provides the mean, SD, minimum, maximum, and the coefficient of variation values of the points of interest in the regular *PPP* and n*PPP* based on 4234 cardiac cycles. By definition, normalization reduced the variation in P_max_, P_min_, +dP/dt_peak_, and –dP/dt_peak_ to 0. The variation of P_C_ (9.6%) changed slightly compared with P_NC_ (10.5%) while the variation P_NR_ (6.6%) decreased compared with P_R_ (11.3%). The variation of both nEDP (27.1%) and nESP (8.9%) increased in comparison to EDP (17.9%) and ESP (6.9%). Among all these P_NR_ had the lowest variation. To illustrate intersubject variation in *PPP* loop shape and features Figure 3A shows individual, superimposed beats from three subjects. Figure 3B shows same beats in the *nPPP*, illustrating the effect of normalization in eliminating the differences in Figure 3A.

**Figure 4:**
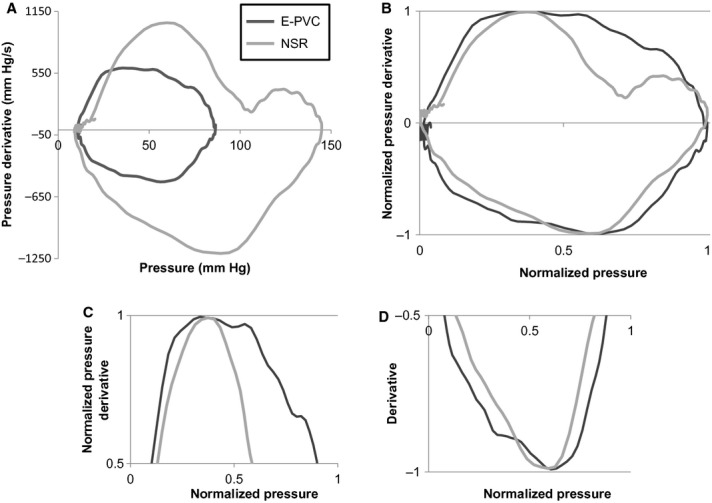
(A) *PPP* from two beats – NSR and E-PVC recorded from Subject B3. (B) Normalization of the same two beats. (C) Magnified view of n*PPP* top portion, including +dP/dt _peak_. (D) Magnified view of n*PPP* bottom portion including –dP/dt _peak_. Data points were smoothed using three point moving average. See text for details.

To investigate *nPPP* features and determine the effect of normalization on P_NR_, we compared NSR beats to E-PVCs and NE-PVCs in the same subject. We selected three datasets that had significant number of PVCs to permit statistical analysis. Table 2 shows the clinical characteristics. Figure 4 shows *PPP* and *nPPP* for a NSR and E-PVC beat in the same subject. For clarity, Figure 4C and D magnifies the P_NC_ and P_NR_ portions of Figure 4B. As these figures illustrate, normalization aids in visualizing features masked by the differences in P_max_, P_min_, +dP/dt_peak_, and –dP/dt_peak_. In Subject B1 (see Table 2) we compared NE-PVCs to NSR beats. In concordance with previous results (Carroll et al. 1983), we found that P_max_, +dP/dt_peak_, and –dP/dt_peak_ were significantly lower in magnitude in the PVC beats (*P* < 0.01). While P_R_ was statistically different between the two types of beats (*P* < 0.001); P_NR_ was not statistically different (*P* = 0.09). In subjects B2 and B3 (Table 2), we compared NSR beats to E-PVC beats but not to NE-PVCs because of limited numbers. Similar to subject B1, we found that in B2 and B3, P_max_, +dP/dt_peak_, and –dP/dt_peak_ were much lower in magnitude in the PVC beats (*P* < 0.0001). Also P_R_ was statistically different between the two types of beats; the value of P_NR_ was not statistically different (*P* = 0.09 and *P* = 1). This suggests that P_NR_ remains essentially unaltered between NSR and PVC beats. The mean value of P_NR_ (0.64) in the three datasets with PVCs was comparable to the value of P_NR_ (0.61) obtained from the first part of the study analyzing 14 datasets. While P_C_ did not differ between NSR and PVC due to large intrasubject variation (12.3%), P_NR_ did not differ in spite of the low beat to beat variation (6.3%) indicating a much smaller distribution of P_NR_ values in NSR and PVC beats. The mean values of the hemodynamic parameters of the *PPP* and the n*PPP* in NSR and PVC beats are given in Table 4 along with measures of statistical significance.

**Table 4:** Mean hemodynamic parameters in NSR versus PVC analysis subjects

	Subject B1		Subject B2		Subject B3	
			
Parameter	NSR	PVC	NSR	PVC	NSR	PVC
P_max_ (mm Hg)	116	101^2^	140	127^2^	132	92^2^
P_min_ (mm Hg)	11	11	12	12	9	8
dP/dt_max_ (mm Hg/sec)	996	758^2^	1190	1026^2^	1007	652^2^
dP/dt_min_ (mm Hg/sec)	−1121	−861^1^	−1169	−1019^2^	−1136	−584^2^
P_C_ (mm Hg)	61	52	73	73	53	47
P_R_ (mm Hg)	73	61^2^	99	88^2^	87	60^2^
P_NC_	0.47	0.45	0.48	0.53^2^	0.36	0.46^1^
P_NR_	0.63	0.59	0.67	0.66	0.63	0.63
EDP (mm Hg)	16	-NA-	16	-NA-	16	-NA-
ESP (mm Hg)	106	-NA-	123	-NA-	89	-NA-

The number of beats is given in Table 2.

1 *P* < 0.01.

2 *P* < 0.0001.

**Table 5:** List of Abbreviations and units of measurement

Abbreviation	Full Term	Unit
*PPP*	Pressure phase plane	-NA-
P	Left ventricular pressure	mm Hg
dP/dt	Time rate of change of LV pressure	mm Hg/sec
P_min_	Minimum LV pressure	mm Hg
P_max_	Maximum LV pressure	mm Hg
+dP/dt_peak_	Peak positive dP/dt	mm Hg/sec
−dP/dt_peak_	Peak negative dP/dt	mm Hg/sec
P_C_	Pressure at +dP/dt_peak_	mm Hg
P_R_	Pressure at –dP/dt_peak_	mm Hg
EDP	End-diastolic pressure	mm Hg
ESP	End-systolic pressure	mm Hg
*nPPP*	Normalized pressure phase plane	-NA-
P_NC_	Normalized pressure at +dP/dt_peak_	Dimensionless
P_NR_	Normalized pressure at −dP/dt_peak_	Dimensionless
nEDP	Normalized end-diastolic pressure	Dimensionless
nESP	Normalized end-systolic pressure	Dimensionless
NSR	Normal Sinus Rhythm	-NA-
E-PVC	Ejecting premature ventricular contraction	-NA-
NE-PVC	Nonejecting premature ventricular contraction	-NA-
IVR	Isovolumic relaxation	-NA-
IVC	Isovolumic contraction	-NA-

## Discussion

Normalization of diastolic physiologic data has been employed in a different context previously. Klotz et al. (2006) proposed a method to estimate the end-diastolic pressure–volume relationships by normalizing LV volumes. They found that normalization generated end-diastolic pressure–volume curves having the same shape across different species and pathologies.

The phase plane method has been used in biological and physiological systems (Paniflov and Hogeweg 1995; Keener and Sneyd 1998). LV pressure phase plane analysis, a component of 4-dimensional physiologic hyperspace (Eucker et al. 2002), has been previously employed (Eucker et al. 2001; Chung et al. 2006; Chung and Kovács 2007) to identify new cardiac cycle features. In the quest to identify load-independent chamber properties we explored LV hemodynamics in the *nPPP*. Normalization maps the variable maximum and minimum pressure and dP/dt limits of consecutive beats to the same values and thereby removes loading effects while contraction and relaxation related loop shape features are retained. We evaluated IVC and IVR loop features in different subjects to characterize differences and similarities. We also compared NSR beats to PVCs in three subjects. Both studies showed that P_NR_ remains essentially invariant while other *nPPP* points of interest varied significantly. Hence, *nPPP* analysis indicates that P_NR_ is closely conserved.

### Load dependence of LV relaxation

Chamber relaxation is known to be determined in part by cross-bridge uncoupling and other load-dependent mechanisms (Katz 1930; Karliner et al. 1977; Brutsaert et al. 1980; Gaasch et al. 1980; Hori et al. 1985; Eichhorn et al. 1992; Chemla et al. 2000; Janssen 2010; Senzaki and Kass 2010). Cross-bridge uncoupling requires dissociation of Ca^2+^ from troponin and its sequestration in the sarcoplasmic reticulum (Bers 2000; Rice and de Tombe 2004). Increased afterload, quantified by end-systolic pressure or volume has a slowing effect on the rate of pressure decay during IVR (Chemla et al. 2000). Other studies have reported that in normal hearts IVR is load independent while failing hearts show increased load sensitivity (Starling et al. 1987; Little 1992; Prabhu 1999). In failing hearts, –dP/dt_peak_ is lower than in normal hearts (Prabhu 1999) and its cause remains uncertain.

### Intersubject comparison of n*PPP*

To determine if *nPPP* can characterize chamber properties that are minimally load dependent or are load independent, we analyzed data from 14 subjects (302 beats/subject, 4234 beats total; Table 1). Normalization eliminated the intersubject variance of P_max_, P_min_, +dP/dt_peak_, and –dP/dt_peak_. Normalization did not alter the variation of pressure at which +dP/dt_peak_ occurs (P_C_ variance = 10% vs. P_NC_ variance = 11%), but it did decrease the variation of pressure at which –dP/dt_peak_ occurs (Table 3) (P_R_ variance = 11%, vs. P_NR_ variance = 6.6%). Thus, in contrast to IVC, normalization generated a much smaller variation in P_NR_ during IVR. This is illustrated in Figure 3 comparing *PPP* (left) and *nPPP* (right) in three selected subjects.

As EDP and ESP depend on preload and afterload, respectively, we normalized these indexes, which generated increased variation in the *nPPP*. The observed increase in variation can be understood by considering at least two effects which influence variation. First, the amplitude of pressure oscillation for each beat, that is, its pressure range and second, the intrinsic contraction/relaxation mechanics. Normalizing removes absolute value effects of pressure without altering intrinsic mechanism effects that determine loop features. Hence the observed increase after normalization suggests that the intrinsic mechanism effects (that determine EDP and ESP) between subjects have higher variation which had been masked by the pressure range variation in *PPP*.

### Hemodynamics of premature ventricular contractions

PVCs provide natural (in contrast to pharmacologic) beat-to-beat load variation. Many studies have utilized PVCs to characterize load effects in contraction and relaxation. Carroll et al. (1983) studied IVR during PVCs and found that PVCs enhance shortening and augment restoring forces producing a smaller end-systolic chamber. PVCs also delay inactivation and prolong relaxation, generating increased values of τ while impairing LV filling (Stoddard et al. 1989). PVCs have also been employed to more extensively validate a load independent index of diastolic function (Boskovski et al. 2008).

### Effect of normalization on PVC hemodynamics

We exploited PVC generated load variation to assess load-dependent features in the *PPP* and the *nPPP*. Information on the PVC datasets is given in Table 2 and Table 4. Figure 4A shows a NSR and E-PVC beat in the *PPP* and Figure 4B shows the same beats in *nPPP*. As seen in the figure, E-PVC has lower values of P_max_, +dP/dt_peak_, and –dP/dt_peak_. P_C_ was not significantly different between NSR and PVC beats although large beat-to-beat variation in each subject was present. P_R_ was significantly lower in PVCs in all the three subjects (*P* < 0.001).

The value of P_NR_ was not significantly different among the three subjects between NSR and PVC beats. This revealed that there is essentially no change in the dimensionless pressure at which the peak rate of pressure decay occurred in both NSR and PVC beats. This value is comparable to the value of P_NR_ obtained from the first part of the study (Table 3, P_NR_ = 0.61). P_NC_ on the other hand was higher in E-PVC compared with NSR. Hence unlike contraction, which shows changes in the rate of pressure rise as a function of pressure in PVCs, the rate of pressure decay as a function of pressure is essentially unchanged during IVR in PVCs suggesting a (relatively) conserved intrinsic relaxation mechanism.

### Physiological significance of normalization and possible mechanism

LV contraction and relaxation involves actin–myosin cross-bridge coupling and uncoupling regulated by Ca^2+^ bound to troponin (Baker et al. 1998; Bombardini 2005; de Tombe et al. 2010) and further modulated by the loading conditions involving pressure and its variation. Studies have attempted to understand the contribution of load as factors in contraction and relaxation (Brutsaert et al. 1980; Hori et al. 1985; Starling et al. 1987; Little 1992; Prabhu 1999) by physiologic, pharmacologic, or surgical interventions to modify load and evaluate response.

Some of the factors determining these intrinsic mechanisms include calcium cycling, sarcomere kinetics, mitochondrial (ATP) function, extracellular matrix, etc. The similar variation of P_C_ and P_NC_ (Table 3) suggests that its variation is determined by factors other than load. On the other hand, the reduced variation of P_NR_ as compared to P_R_ suggests that its variation is load dependent but the intrinsic mechanism that constrains P_NR_ to be in the 0.61–0.64 range is conserved. This underscores that *nPPP* is not merely a scaled down version of the regular *PPP*. Rather normalization removes P and dP/dt magnitude effects while maintaining shape-based features.

Maintenance of shape-based features was borne out by the intrasubject PVC analysis. The value of P_NR_ was similar in NSR and PVC beats within and across subjects (Table 4). Its value was also similar to the value reported in the intersubject analysis (Table 3). This permits the inference that intrinsic relaxation mechanisms are more (tightly regulated) conserved than intrinsic contraction mechanisms. Moreover, contractility and the associated value of P_NC_ in NSR and E-PVC beats is governed by the beat-to-beat variation of preload and afterload while in NE-PVCs it is primarily determined by the Frank–Starling Law and the timing of the PVC relative to the prior beat. Hence, an *nPPP* based prediction is that because of the beat-to-beat variation of load, we expect P_NC_ to have larger variation than P_NR_. Our observations corroborate this prediction. Sarcomere kinetics is a major determinant of relaxation mechanisms (Piroddi et al. 2007; Stehle et al. 2009). Two features of sarcomere kinetics likely to have a bearing on the limited variation of P_NR_ include (Little et al. 2012) – (1) kinetics of Ca^2+^ binding and dissociation from troponin and (2) cross-bridge attachment/detachment and subsequent sarcomere shortening/lengthening. The relative constancy of P_NR_ suggests that the ratio of maximum rate of cross-bridge dissociation to instantaneous pressure (force, wall stress) at which that maximum dissociation rate takes place is tightly constrained. This preliminary, proof of concept study demonstrates the utility of *PPP* normalization in elucidating novel LV diastolic properties.

### Limitations

The main limitations pertain to data acquisition. As noted previously (Ghosh and Kovács 2012), calibration, catheter placement, and orientation with respect to the LV axis may have a slight effect on pressure recordings. However, calibration offsets the pressure by a constant value which should not affect the normalization process. Calibration and drift are mitigated by pre- and postcalibration of transducers to zero hydrostatic pressure in a 37°C saline bath. Other issues involving signal processing have been addressed previously (Chung and Kovács 2008; Shmuylovich and Kovács 2008; Ghosh and Kovács 2012). Noisy beats were not analyzed. Moreover, the large (average) number of beats studied in every subject (302) mitigates the effect of noise to an acceptable degree.

As this is a proof of concept study, the number of datasets analyzed is necessarily limited although the 4234 cardiac cycles analyzed mitigates that limitation to an acceptable degree. Although eight of the datasets analyzed in this study have been previously analyzed for different purposes (16), repeat analysis using a different method (normalization) to test a different hypothesis (load independence of phase plane loop features) is appropriate. Relative physiologic uniformity is achieved as a result of enrollment criteria (normal ejection fraction, no coronary artery disease or myocardial infarctions, no diabetes). P and dP/dt values were not very different (<50% variation in P_max_, +dP/dt_peak_, P_C_, and P_R_ values). This limitation is mitigated by the second part of the study where we compared NSR to PVC beats in the same subject. The *PPP* in PVC is much smaller and has a different shape from a NSR *PPP* (Fig. 3; Chung and Kovács 2008). In spite of this, P_NR_ remained an essentially conserved feature among the three subjects. However, we only studied PVCs in three subjects, which is insufficient to draw definitive conclusions regarding trends. Hence, additional studies are needed to elucidate the magnitude of these changes and differentiate between the changes in E-PVCs versus NE-PVCs. Further work in the *PPP* and physiologic hyperspace is needed involving a greater sample size and specific pathophysiologic states.

## Conclusions

We introduce the *nPPP* for LV hemodynamic analysis. Normalization removes beat-to-beat and intersubject variation in P and dP/dt limits and thereby, minimizes load effects. We tested applicability in ∼4400 beats in 14 subjects. In the *nPPP*, the variation of P_NR_, the (dimensionless) pressure at which –dP/dt_peak_ was inscribed, was very substantially reduced. Comparison of NSR beats to both ejecting and nonejecting beats PVCs revealed that P_NR_ remained tightly controlled. The observed near constancy of P_NR_ reveals a new aspect of the physiology of diastole and indicates the existence of intrinsic (intracellular) IVR mechanisms for which a possible mechanism is discussed. Thus, *nPPP* analysis elucidates novel LV chamber properties, and identifies potential research targets in need of molecular and cellular physiologic explanation.

## References

[b1] Baker AJ, Figueredo VM, Keung EC, Camacho SA (1998). Ca^2+^ regulates the kinetics of tension development in intact cardiac muscle. Am J Physiol Heart Circ Physiol.

[b2] Bers DM (2000). Calcium fluxes involved in control of cardiac myocytes contraction. Circulation.

[b3] Bombardini T (2005). Myocardial contractility in the echo lab: molecular, cellular and pathophysiological basis. Cardiovasc Ultrasound.

[b4] Boskovski M, Shmuylovich L, Kovács SJ (2008). Transmitral flow velocity-contour variation after premature ventricular contractions: a novel test of the load-independent index of diastolic filling. Ultrasound Med. Biol.

[b5] Brutsaert DL, Housmans PR, Goethals MA (1980). Dual control of relaxation. Its role in the ventricular function in the mammalian heart. Circ. Res.

[b6] Carroll JD, Widmer R, Hess OM, Hirzel HO, Krayenbuehl HP (1983). Left ventricular isovolumic pressure decay and diastolic mechanics after postextrasystolic potentiation and during exercise. Am. J. Cardiol.

[b7] Chemla D, Coirault C, Hébert J, Lecarpentier Y (2000). Mechanics of relaxation of the human heart. News Physiol. Sci.

[b8] Chung CS, Kovács SJ (2007). Pressure Phase-Plane Based Determination of the Onset of Left Ventricular Relaxation. Cardiovasc. Eng.

[b9] Chung CS, Kovács SJ (2008). The physical determinants of left ventricular isovolumic pressure decline: model-based prediction with in-vivo validation. Am J Physiol Heart Circ Physiol.

[b10] Chung CS, Strunc A, Oliver R, Kovács SJ (2006). Diastolic ventricular-vascular stiffness and relaxation relation: elucidation of coupling via pressure phase plane-derived indexes. Am J Physiol Heart Circ Physiol.

[b11] Eichhorn EJ, Willard JE, Alvarez L, Kim AS, Glamann DB, Risser RC (1992). Are contraction and relaxation coupled in patients with and without congestive heart failure?. Circulation.

[b12] Eucker SA, Lisauskas JB, Singh J, Kovács SJ (2001). Phase Plane Analysis of Left Ventricular Hemodynamics. J. Appl. Physiol.

[b13] Eucker SA, Lisauskas J, Courtois MR, Kovács SJ (2002). Analysis of left ventricular hemodynamics in physiological hyperspace. J. Appl. Physiol.

[b14] Gaasch WH, Blaustein AS, Andrias W, Donahue RP, Avitall B (1980). Myocardial relaxation II. Hemodynamic determinants of rate of left ventricular isovolumic pressure decline. Am J Physiol Heart Circ Physiol.

[b15] Ghosh E, Kovács SJ (2012). Spatio-temporal attributes of left ventricular pressure decay rate during isovolumic relaxation. Am J Physiol Heart Circ Physiol.

[b16] Hori M, Inoue M, Kitakaze M, Tsujioka K, Ishida Y, Fukunami M (1985). Loading sequence is a major determinant of afterload- dependent relaxation in intact canine heart. Am J Physiol Heart Circ Physiol.

[b17] Janssen PML (2010). Myocardial contraction- relaxation coupling. Am J Physiol Heart Circ Physiol.

[b18] Karliner JS, Lewinter MM, Mahler F, Engler R, O'Rourke RA (1977). Pharmacologic and hemodynamic influences on the rate of isovolumic left ventricular relaxation in the normal conscious dog. J Clin Invest.

[b19] Katz LN (1930). The role played by the ventricular relaxation process in filling the ventricle. Am. J. Physiol.

[b20] Keener J, Sneyd J (1998). Mathematical Physiology: interdisciplinary Applied Mathematics.

[b21] Klotz S, Hay I, Dickstein ML, Yi GH, Wang J, Maurer MS (2006). Single-beat estimation of end-diastolic pressure-volume relationship: a novel method with potential noninvasive application. Am J Physiol Heart Circ Physiol.

[b22] Leite-Moreira AF, Correia-Pinto J, Gillebert TC (1999). Load dependence of left ventricular contraction and relaxation. Effects of caffeine. Basic Res. Cardiol.

[b23] Little WC (1992). Enhanced load dependence of relaxation in heart failure. Clinical implications. Circulation.

[b24] Little SC, Biesiadecki BJ, Kilic A, Higgins RSD, Janssen PML, Davis JP (2012). The rates of Ca^2+^ dissociation and cross-bridge detachment from ventricular myofibrils as reported by a fluorescent cardiac Troponin C*. J. Biol. Chem.

[b25] Matsubara H, Takaki M, Yasuhara S, Araki J, Suga H (1995). Logistic time constant isovolumic relaxation pressure- time curve in the canine left ventricle. Circulation.

[b26] Paniflov AV, Hogeweg P (1995). Spiral break-up in a modified FitzHugh- Nagumo model. Phys. Lett. A.

[b27] Piroddi N, Belus A, Scellini B, Tesi C, Giunti G, Cerbai E (2007). Tension generation and relaxation in single myofibrils from human atrial and ventricular myocardium. Eur J Physiol.

[b28] Prabhu SD (1999). Load sensitivity of left ventricular relaxation in normal and failing hearts: evidence of a nonlinear biphasic response. Cardiovasc. Res.

[b29] Rice JJ, de Tombe PP (2004). Approaches to modeling crossbridges and calcium- dependent activation in cardiac muscle. Prog. Biophys. Mol. Biol.

[b30] Senzaki H, Kass DA (2010). Analysis of isovolumic relaxation in failing hearts by monoexponential time constants overestimates lusitropic change and load dependence. Circ Heart Fail.

[b31] Shmuylovich L, Kovács SJ (2008). Stiffness and relaxation components of the exponential and logistic time constants may be used to derive a load-independent index of isovolumic pressure decay. Am J Physiol Heart Circ Physiol.

[b32] Starling MR, Montgomery DG, Mancini GBJ, Walsh RA (1987). Load independence of the rate of isovolumic relaxation in man. Circulation.

[b33] Stehle R, Solzin J, Iorga B, Poggesi C (2009). Insights into the kinetics of Ca2+- regulated contraction and relaxation from myofibril studies. Eur J Physiol.

[b34] Stoddard MF, Pearson AC, Kern MJ, Labovitz AJ (1989). The effect of premature ventricular contraction on left ventricular relaxation, chamber stiffness and filling in humans. Am. Heart J.

[b35] Strogatz SH (2008). Non-linear dynamics and chaos.

[b36] de Tombe PP, Mateja RD, Tachampa K, Mou YA, Farman GP, Irving TC (2010). Myofilament length dependent activation. J. Mol. Cell. Cardiol.

[b37] Weiss JL, Frederiksen JW, Weisfeldt ML (1976). Hemodynamics determinants of the time course of fall in canine left ventricular pressure. J Clin Invest.

